# Effect of CYP2D6, 2C19, and 3A4 Phenoconversion in Drug-Related Deaths

**DOI:** 10.3390/toxics12040260

**Published:** 2024-03-30

**Authors:** Sanaa M. Aly, Benjamin Hennart, Jean-Michel Gaulier, Delphine Allorge

**Affiliations:** 1 Forensic Medicine and Clinical Toxicology Department, Faculty of Medicine, Suez Canal University, Ismailia 41522, Egypt; 2 CHU Lille, Service de Toxicologie-Génopathies, F-59000 Lille, France; 3 ULR 4483—IMPECS—IMPact de l’Environnement Chimique sur la Santé Humaine, Université de Lille, F-59000 Lille, France

**Keywords:** genotype–phenotype mismatch, pharmacogenetics, cytochrome P450, drug-gene interaction, drug-metabolizing enzymes, personalized medicine

## Abstract

Molecular autopsy is a very important tool in forensic toxicology. However, many determinants, such as co-medication and physiological parameters, should be considered for optimal results. These determinants could cause phenoconversion (PC), a discrepancy between the real metabolic profile after phenoconversion and the phenotype determined by the genotype. This study’s objective was to assess the PC of drug-metabolizing enzymes, namely CYP2D6, 2C19, and 3A4, in 45 *post-mortem* cases where medications that are substrates, inducers, or inhibitors of these enzymes were detected. It also intended to evaluate how PC affected the drug’s metabolic ratio (MR) in four cases. Blood samples from 45 cases of drug-related deaths were analyzed to detect and determine drug and metabolite concentrations. Moreover, all the samples underwent genotyping utilizing the HaloPlex Target Enrichment System for *CYP2D6*, *2C19*, and *3A4*. The results of the present study revealed a statistically significant rate of PC for the three investigated enzymes, with a higher frequency of poor metabolizers after PC. A compatibility was seen between the results of the genomic evaluation after PC and the observed MRs of venlafaxine, citalopram, and fentanyl. This leads us to focus on the determinants causing PC that may be mainly induced by drug interactions. This complex phenomenon can have a significant impact on the analysis, interpretation of genotypes, and accurate conclusions in forensic toxicology. Nevertheless, more research with more cases in the future is needed to confirm these results.

## 1. Introduction

Genetic polymorphisms modulate human cytochrome P450 (CYP) drug-metabolizing enzymes (DMEs). This polymorphism is responsible for genotypes with the corresponding genotype-predicted phenotype (g-phenotype) that are categorized as ultrarapid (gUM), normal (gNM), intermediate (gIM), and poor (gPM) metabolizers [1]. The differences in drug responses are explained by this inter-genotypic heterogeneity [2]. Twenty to ninety-five percent of the diversity in drug responses and effects is thought to be caused by variations in the genes encoding DMEs, drug transporters, or drug targets [3]. The discrepancy between a g-phenotype and an individual’s actual ability to metabolize drugs owing to non-genetic variables is known as phenoconversion (PC). These variables may include age, weight, sex, diseases, food, and concurrent drug use. Taking drugs that modify effects (inhibition or induction) on DMEs may result in drug-drug interactions (DDIs) and drug–drug–gene interactions (DDGIs) [4,5]. It is noteworthy to mention that the inhibitory strength of some substances, whether strong or weak (e.g., olanzapine, cocaine, and lidocaine), is either controversial in the literature or only in vitro data are available. This limitation is problematic in evaluating toxicity cases and in forensic toxicogenetics [6].

Personalized medicine gives physicians more accurate tools to choose a treatment plan based on a comprehensive approach that potentially reduces adverse drug reactions (ADRs) and provides a safe and successful outcome [7]. This approach takes additional non-genetic factors and DNA-based phenotypes into account. This could potentially aid in the prevention of ADRs, which rank among the top ten most common causes of death in developed countries [8]. Each genotype may have a different PC impact. For instance, gIM may develop into a PM if an enzyme inhibitor is used concurrently. Since there is no enzyme to be inhibited in PMs, such cases may not be affected by the same enzyme inhibitor administered concurrently. Greater plasma concentrations of gIM and gPM in a clinical context raise the possibility of harm. However, individuals with gUM may be at risk of therapeutic failure due to excessive metabolism [9]. Consequently, a mismatch between genotype and phenotype may have serious implications in clinical settings and lead to patients receiving inadequate therapy [4,10].

In forensics, the term “molecular autopsy” refers to a medico-legal death investigation that includes genetic testing. It may be especially helpful in cases of unexpected sudden deaths or drug-related deaths [11,12,13]. In fact, the toxicogenetics method could be useful in toxicological result interpretations. It has been confirmed that pharmacogenetics has shown its utility in understanding or even solving a number of difficult lethal intoxicated cases [13]. The approach used is still primarily focused on DNA-based phenotypes, which may not alone be optimal for toxicological interpretation. Toxicological conclusions may be impacted by phenotype and other variables like co-medication or physiological characteristics.

In this situation, this study’s objective was to assess the PC of DMEs, namely CYP2D6, 2C19, and 3A4, in 45 *post-mortem* cases where medications that are substrates, inducers, or inhibitors of these enzymes were detected. It also intended to evaluate how PC affected the drug’s metabolic ratio (MR) in 4 cases (out of 45 cases in which parent drugs and their metabolites were detected as well).

## 2. Materials and Methods

### 2.1. Sample Collection

Forty-five cases were included in this study. Femoral blood samples were collected during autopsies. The sample collections were performed according to previously published guidelines [14]. Inclusion criteria were as follows: (1) cases submitted after a complete autopsy at the Toxicological Unit of the University Hospital of Lille (North of France) between 2013 and 2020; (2) toxicological analysis and tested positive for drugs and psychotropic substances metabolized by, or inhibitors/inducers of, CYP2D6, 2C19, and 3A4. Exclusion criteria were cases with late *post-mortem* changes, undetermined cause (CoD) and mode (MoD) of death, unsuccessful DNA extraction, or a sample with a low amount that was insufficient for further analyses. When the specimens were collected during autopsy, they were directly kept in storage at −20 °C. Anonymized data were collected, including age, gender, past history, drug use, diseases, CoD and MoD, toxicological results, and genetic analysis.

### 2.2. Toxicological Analysis

A thorough toxicological evaluation was conducted for each case, including general screenings of peripheral blood (10 mL of femoral blood) [15] using previously published liquid chromatography using a high-resolution mass spectrometry approach [16,17,18]. Liquid chromatography coupled to tandem mass spectrometry detection (LC-MS/MS) or diode array detection (LC-DAD) and headspace-gas chromatography with flame ionization detection (for volatiles, including alcohols) were used to carry out more selective assays for multiple classes of therapeutic drugs, drugs of abuse, or other toxicants using various ad-hoc dedicated methods.

### 2.3. CYP2D6, CYP2C19, and CYP3A4 Genotyping

To provide the genotype assessment for CYP2D6, 2C19, and 3A4, 1 mL of whole blood from each sample was extracted using a Robot DNA Extractor Chemagic-Star (Hamilton Company, Reno, NV, USA) and a Perkin-Elmer/B2K extraction kit (Perkin-Elmer, Waltham, MA, USA). Every DNA extract was adjusted to a final 5 ng/μL concentration. A NanoDrop One/OneC UV–visible microvolume spectrophotometer (Thermo Scientific, Schwerte, Germany) was used to assess the quality and amount of isolated DNA.

The chosen markers were mapped and evaluated in relation to the HaloPlex Target Enrichment System’s known restriction enzyme cleavage sites (Table 1). The HaloPlex Target Enrichment System (Agilent Technologies, Santa Clara, CA, USA) was used to prepare each run of all samples and two positive controls (Enrichment Control DNA, given by Agilent), in accordance with the manufacturer’s methodology (protocol version G 0; Agilent Technologies Inc., Mumbai, Maharashtra). To amplify the target libraries that were captured, an Applied Biosystems GeneAmp PCR System 9700 (Thermo Fisher Scientific, Waltham, MA, USA) was used for thermal cycling. Following the manufacturer’s instructions, the Agilent 4200 Tape Station (Agilent Technologies, Mumbai, Maharashtra, India) was used to quantify the enriched target DNA in each library sample using the Agilent High Sensitivity D1000 Screen Tape. Using the NextSeq 550 (Illumina Inc., San Diego, CA, USA), sequencing was performed. In accordance with the NextSeq 550 System User Guide (Illumina Inc., San Diego, CA, USA), the enrichment libraries were combined and diluted [19].

Using the QuantStudio^TM^ RT-PCR software V1.3 (Applied Biosystems, Waltham, MA, USA), a TaqMan^®^ Copy Number Assay (Thermo Fisher Scientific Inc., Waltham, MA, USA) was used to determine the number of copies of the *CYP2D6* gene. The reaction mixture consisted of 12.5 μL Brilliant II SYBR Green qPCR master mix, 0.125 μL of each 100 μM primer, 7.5 μL H_2_O, and DNA extracts with different concentrations (2, 1, 0.2, and 0.02 ng/μL). For genotyping analysis, the following PCR conditions were used: an initial step at 50 °C for 2 min and an initial denaturation and enzyme activation step at 95 °C for 10 min, followed by 40 cycles at 95 °C for 15 s and at 60 °C for 1 min. An assay for the Ribonuclease P RNA component H1 gene (*RNase P*, assay ID 4403326) was used as a reference to determine the copy number. For each run of samples, samples carrying one and three copies were included in all plates as CYP2D6 copy number controls [16].

### 2.4. Assessment of Phenotypes

An activity score (AS) was assigned in order to evaluate the phenotype of *CYP2D6* based on its genotype [16]. The phenotypic attribution for *CYP2D6*, *2C19*, and *3A4* was determined using PHARMGKB tables [20]. To differentiate the phenotypic categories resulting from PC from those based on the real genotype (g-phenotype), the term “p-phenotype” was employed [21].

The assessment of CYP2D6, 2C19, and 3A4 inducers and inhibitors was carried out [6]. The values of the AS were modified in accordance with the guidelines provided by Borges et al. [22], which introduced the use of inhibition factors (e.g., multiplication of the AS by 0 in the case of a strong inhibitor and by 0.5 in the case of a weak or moderate inhibitor) in order to assess the p-phenotypes of *CYP2D6* [23]. For CYP2D6, the starting ASs of different g-phenotypes were assessed as gPM (0), gIM (0 < x < 1.25), gNM (1.25 ≤ x ≤ 2.25), and gUM (>2.25). In the case of strong inhibitor administration, the AS was multiplied by 0. Thus, any adjusted AS will be 0 and the p-phenotype will be pPM. In the case of a moderate or weak inhibitor, the AS was multiplied by 0.5. Thus, the adjusted AS will be reduced to half. For example, the case with an AS of 1.25 ≤ x ≤ 2.25 (gNM) will be with an adjusted AS at 0 < x < 1.25 (pIM) with moderate inhibitor administration. For *CYP2C19* and *3A4,* the p-phenotypes were modified in accordance with Mostafa et al.’s study [5]. The g-phenotypes were assessed as gPM, gIM, gNM, and gUM. In cases of strong, moderate, or two weak inhibitor administrations, gNM and gIM will be pPM and gUM will be pIM.

### 2.5. Statistical Analyses

Descriptive statistics were conducted for all the data. Male and female age differences were tested using an ANOVA (analysis of variance) test. The mean and standard deviation (SD) of the blood levels were computed in order to quantify drugs of abuse. The CoD was used to divide the cases into three groups: (1) non-intoxication, which occurs when the CoD was any cause of death but not fatal intoxication; (2) mono-intoxication, which occurs when only one substance was recovered as the CoD; (3) mixed intoxication, which occurs when many substances contributed to the toxicity. Chi-square analysis was used to evaluate the following: (A) the associations between categorical variables (gender, CoD, or MoD); (B) the relationships between categorical variables, such as g-phenotype vs. p-phenotype, to evaluate whether PC affected the frequency of PM, IM, NM, and UM; and (C) the distribution of p-phenotypes in the three CoD groups and in the MoD groups. Using the statistical software for social sciences (SPSS version 22), statistical tests were run, and *p*-values less than 0.05 were deemed significant.

## 3. Results

The mean age of the 45 cases in the present study was 55.8 years (standard deviation: 19.4), with a range of 18 to 91 years. About 15.5% of cases were between the ages of 18 and 35, 28.9% were between the ages of 36 and 50, 28.9% were between the ages of 51 and 65, and 26.7% were older than 65. Women made up 16 of the deceased, or 35.6% of the total. For mean age, there was no statistical difference between men and women (*p* = 0.28). CoD was divided into three categories: non-intoxications (25%), mixed intoxications (24.4%), and mono-intoxications (20%). Within the CoD groups, there was no statistically significant difference (*p* > 0.05) in the distribution of sex. In 77.8% of cases, MoD was categorized as accidental, in 11.1% as suicide, and in 11.1% as other. There were statistically significant disparities (*p* < 0.05) in the distribution of sex among the MoD groups (Table 2). Two cases (4.4%) had a history of cancer, four cases (8.9%) had psychiatric diseases, one case (2.2%) had heart disease, seven cases (15.6%) had other diseases, and some of these conditions were in combination.

Methadone was detected in one case, and alcohol was detected in nine cases, ranging from 0.11 to 3 g/L. Two subjects tested positive for morphine. Cocaine was detected in two cases. Benzodiazepines were detected in 14 subjects, antipsychotics were detected in 9 cases, opioid medications were detected in 3 cases (fentanyl and codeine), and paracetamol was detected in 19 cases.

### 3.1. CYP2D6, 2C19, and 3A4 Genotype and g-Phenotype

Genotyping for the three tested DME genes was successfully performed for all samples. Table 3 shows *CYP2D6*, *2C19*, and *3A4* allele functions identified in the present study. Table 4 shows the frequencies of genotypes and the g-phenotypes of all the samples.

Five subjects (11%) were *CYP2D6* gPM with no function alleles (*3, *4, or *6), including two subjects that had a deletion of the other allele (*5). The *CYP2C19* gPM (31.1%) group showed two no-function alleles (*2 and *35). No subject is classified as *CYP3A4* gPM. The frequencies of *CYP2D6*, *2C19*, and *3A4* gIMs were 22.2%, 68.9%, and 15.6%, respectively. Regarding the NM g-phenotype, there were 27 subjects (60%) for *CYP2D6*, no subject for *CYP2C19*, and 38 subjects (84.4%) for *CYP3A4*. The three subjects (6.7%) classified as *CYP2D6* gUMs harbored *1/*1 × 2 and *4/*1 × 3 gene duplications.

### 3.2. PC and DGIs

The substances that were detected in the present study with inhibitory effects against CYP2D6 are as follows: venlafaxine, tetrahydrocannabinol (9-THC), methadone, clozapine, citalopram, and amiodarone (with moderate/weak inhibition), in addition to cocaine, fluoxetine, and paroxetine (with strong inhibition). Olanzapine and lidocaine were considered weak inhibitors [5]. Amiodarone, citalopram, diazepam, olanzapine, and nordiazepam, with weak/moderate inhibitory effects against CYP2C19, were detected [5]. Finally, the substances that were detected in the present study with inhibitory effects against CYP3A4 are as follows: paroxetine [24] and 9-THC [25] (with strong inhibition). Amiodarone [26] and sildenafil were considered moderate/weak inhibitors. Zolpidem produced a negligible/weak inhibitory effect against CYP2C19, 2D6, and 3A [27]. In the present study, we applied a conservative approach (safety-oriented), using the least effect of the inhibitor that was reported in the literature [6].

**Figure 1:**
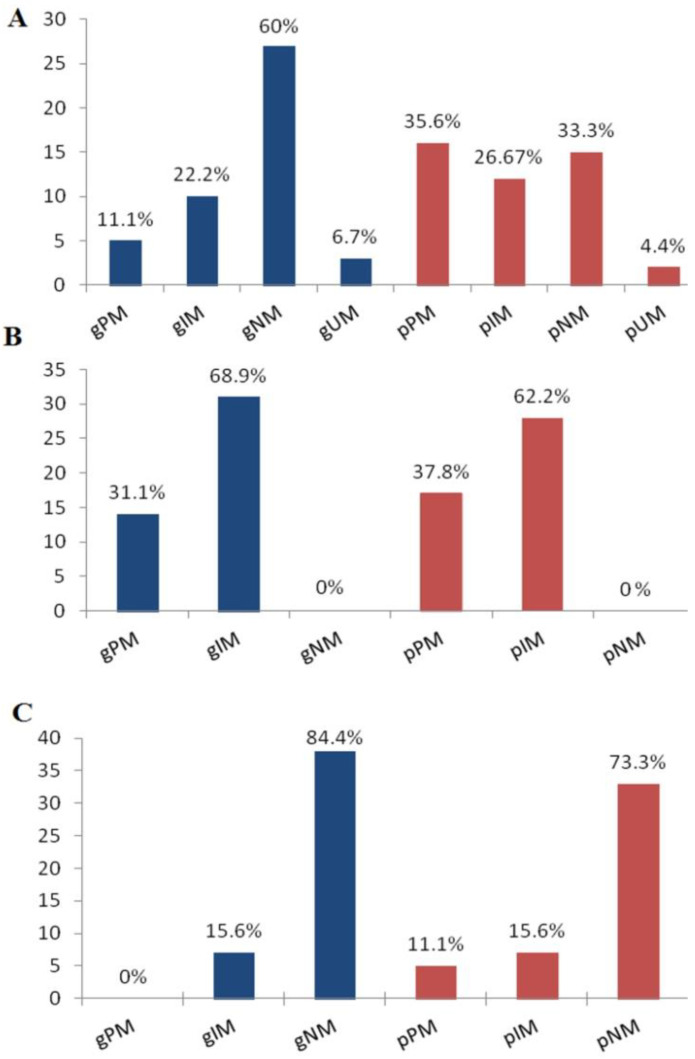
Distributions of *CYP2D6* (**A**), *2C19* (**B**), and *3A4* (**C**) g-phenotypes and p-phenotypes. g-: phenotype based on genotype; p-: phenotype after phenoconversion; PM: poor metabolizer; IM: intermediate metabolizer; NM: normal metabolizer; and UM: ultrarapid metabolizer.

In Figure 1, the g-phenotype distributions with corresponding p-phenotypes for the three analyzed DMEs are reported. For *CYP2D6*, *2C19*, and *3A4,* the relationships between the g-phenotype categories (gPM, gIM, gNM, and gUM) and p-phenotypes were statistically significant (*p* < 0.001). For the three DMEs under evaluation, the distribution of p-phenotypes among the CoD and MoD groups did not exhibit a statistically significant correlation (*p* > 0.05).

### 3.3. PC Effect on MR of the Detected Drugs

Among the cases described here, the parent drugs and their main metabolites were detected in the blood samples of four cases. A 66-year-old female was known to have cancer. A toxicological blood analysis showed the following drugs and concentrations in µg/L: paracetamol 1480, venlafaxine (VEN) 130, o-desmethylvenlafaxine (ODV) 256, oxazepam 86, zolpidem 932, and tramadol 231 (Table 5, *case #1*). A 90-year-old male was dead in the hospital. The fentanyl was given by the physician in the hospital. A toxicological blood analysis showed the following drugs and concentrations in µg/L: venlafaxine (VEN) 13, o-desmethylvenlafaxine (ODV) 19.7, slidenafil 92, fentanyl (Fen) 5, and nor-fentanyl (Nfen) 0.6 (Table 5, *case #2*).

A 36-year-old female was found dead. A toxicological blood analysis showed the following drugs and concentrations in µg/L: citalopram 2145, N-desmethylcitalopram (DCT) 359, 9-THC 23.5, and 9-THC-COOH 45 (Table 5, *case #3*). A 37-year-old male was found dead. He was on baclofen as a treatment for alcohol dependence. A toxicological blood analysis showed the following drugs and concentrations in µg/L: methadone 252, 2-ethylidene-1,5-dimethyl-3,3-diphenylpyrrolidene (EDDP) 45, paracetamol 1850, paroxetine 49, baclofen 2224, diazepam 15, and nordiazepam 41 (Table 5, *case #4*)**.**

## 4. Discussion

The value of *post-mortem* pharmacogene genotyping as a supplemental analysis has been the subject of numerous studies to date, but only two previous studies focused on the PC phenomenon in a forensic toxicology context [6,28]. The first study evaluated PC in one case of acute venlafaxine intoxication, showing a discrepancy between the results of pharmacogenetics and the phenotype prediction based on MRs [28]. The second study evaluated PC in 35 cases with positive drugs but without studying the effect on MRs [6]. The present study aimed to assess the PC and its effect on MRs in drug-related deaths. In the present study, 45 cases were typed for *CYP2D6*, *2C19*, and *3A4* gene polymorphisms. These cases were positive for substances metabolized or inhibited by CYP2D6, 2C19, and 3A4. Based on genotyping, the phenotypic groups (gUM, gNM, gIM, and gPM) were classified, then identification of the phenotype was conducted after PC.

By investigating possible relevant factors for PC, there is a significant association between MoD and gender. Males are dominant in accidental deaths, whereas females are dominant in suicidal deaths. A previous study did not find statistical significance between these two factors [6]. Another study agrees relatively well with our finding, as it found that gender distributions were significantly different by MoD, and males were the most represented in all MoD categories [29]. Our result is also in accordance with a previous study that declared that women had 1.78 more suicidal attempts than men [30].

Most of the *CYP2D6* allele frequencies in our investigation agreed with the rates found in Caucasians [31]. The most prevalent *CYP2D6* alleles were CYP2D6*1 and CYP2D6*4, which occurred at frequencies of 71% and 17.7%, respectively, in line with previous reports [5,32]. With a frequency of 4.4%, CYP2D6*1XN was the most prevalent increased function allele among the *CYP2D6* duplicated alleles. In our analysis, the frequency of CYP2C19*1 was lower than the frequencies reported in Caucasians (34.4% vs. 49.2%, respectively) [33]. In contrast, Mostafa et al.’s frequency (39.7%) was comparable to that of the current study [5]. The CYP2C19 enzyme function was altered in 65.6% of the remaining cases, indicating altered drug metabolism of CYP2C19 substrates [5]. The estimated CYP3A4*1 allelic frequency was 92.2%, which is comparable to that previously reported (91.3%) in Caucasians [34]. The estimated allelic frequency of CYP3A4*22 was 7.7%, which is similar to earlier reports in Caucasians (5.6–6.1%) [34,35].

Based on the genotype-predicted phenotype frequencies, a considerable number of the included cases were gPMs (for CYP2D6 and CYP2C19), which would seem to display compromised metabolizing capacity for the analyzed DMEs with a higher probability of drug toxicity. This result is in harmony with the *post-mortem* cases that were presented for forensic toxicology investigation. On the other side, a previous study found just one gPM case (for CYP2D6 and 2C19) [6]. The risk of ADRs could be increased after PC with the inflation of PM frequency due to the co-administration of moderate to strong inhibitors, which probably led to negative outcomes [21]. This is in line with our results, as there was a higher frequency of PM after PC.

In the present study, the frequencies of metabolic classes (gUM, gNM, gIM, and gPM) of *CYP2D6*, *CYP2C19*, and *CYP3A4* were significantly influenced after PC. This is consistent with Storelli et al.’s [36] finding that gNM cases had a higher chance of PC to be pIMs or pPMs due to a moderate or strong inhibitor. Thus, when a certain case has a known CYP genotype and is taking substrate drugs, it is prudent for the treating clinician or the forensic toxicologist to be aware of PC. These instances may have been incorrectly classified, based only on genetic information, as having a low risk of changed drug metabolism. The co-administration of enzyme substrate, inhibitor, or inducer drugs should be kept in mind [5].

In the present study, other factors were considered for PC. There were two cases with cancer. There is proof that pathological conditions [37] significantly affect CYP enzyme activity, which was in accordance with our results. Liver disease and cancer are important causes of the PC of NMs to PMs for CYP2C19 [38,39]. Variations in the *CYP2D6* genotype-phenotype have previously been reported in patients with lung cancer [40]. The toxicological analysis in the present study included analytical quantification methods to cover the full spectrum of metabolites alongside their parent drugs; therefore, MRs were achieved in four cases out of the total. This enabled us to study the effect of PC on MRs and compare it with available data in the literature. The following precautions were taken to avoid the effect of *post-mortem* redistribution (PMR) on the concentration of the parent drugs and metabolites: (1) cases with late *post-mortem* changes were excluded; (2) samples were collected as soon as possible to minimize time passed since death; (3) femoral blood was collected as it is preferred because it is less susceptible to PMR; (4) the samples were stored at −20 °C directly after collection to avoid any change in drug concentration. Although the previously mentioned precautions were taken, other factors could lead to this phenomenon, such as the route of drug administration, *post-mortem* body movement, and the body’s position [41]. Thus, one limitation of this study was PMR.

Venlafaxine (VEN) is a serotonin and noradrenaline reuptake inhibitor that is prescribed for the treatment of depression [42]. Numerous fatalities linked to VEN have been documented [43]. VEN is mainly eliminated through hepatic metabolism, which is mediated by the CYP enzyme system. All CYP2C19, 2D6, and 3A4 isoforms have been linked to VEN metabolism in vitro [14]. These isoforms have been shown to be essential for in vivo VEN elimination [44]. The vast inter-individual heterogeneity in VEN metabolism is primarily due to genetic origins [44,45]. The metabolic capabilities appear to be connected with the significant polymorphic genetic variability exhibited by *CYP2D6* and *CYP2C19* [46]. The VEN case in the present study (*case #1*) displayed that her genotyping of *CYP2D6* and *2C19* were gIMs and became pPMs due to disease-induced phenoconversion (cancer) [27]. The O-desmethylvenlafaxine (ODV) to VEN ratio was found to be significantly lower (0.5) than what would be predicted in a person with a normal metabolic profile, which reflects the enzymatic activity. In this instance, this aberration was caused by PC rather than a genetic variation of the enzyme that impacts function [44,47]. The current study’s genotyping after the PC result is consistent with the findings of Nichols et al. [48], who established that the ODV/VEN ratio discriminated between the phenotypes of EMs and PMs; ratios were larger than or equal to 1 for EMs and less than 1 for PMs. This is also in line with the ratios reported by Kingback et al. [49] (median ratio of 0.23, range 0.08–0.40, n = 6) and Shams et al. [50] (mean ratio of 0.25, n = 4).

*N*-(1-phenylethyl-4-piperidyl) propionanilide, also known as fentanyl, is approximately 80 times more powerful than morphine. It is frequently used to induce and maintain sedation, anesthesia, and analgesia. N-dealkylation quickly converts fentanyl to nor-fentanyl. The ratios of fentanyl to nor-fentanyl in 95% of positive serum or plasma specimens were less than 0.5 [51]. Yuan et al. revealed that there is a positive correlation between the CYP3A4 mRNA level and the metabolism of fentanyl [52]. Barrat et al. showed that the patient with CYP3A4*22 (reduced activity and metabolism) or with CYP3A4 inhibitor co-administration was associated with decreased serum nor-fentanyl concentrations and, consequently, this affected the MR. Conversely, CYP3A4 inducer (including steroids) co-administration was also associated with increased serum nor-fentanyl concentrations. These factors account for only a small proportion of the variability (<2%) in the fentanyl MR [53]. The previously mentioned study agrees with our results. The fentanyl MR in our case (*case #2*) is higher than that documented before in a *post-mortem* setting with molecular autopsy. This difference accounted for the difference in the metabolic state of different cases. The previous case was gUM for CYP3A4, while in our case it was pIM. It is expected that the slow metabolism of fentanyl increased the MR (increase fentanyl in relation to nor-fentanyl) in our case, and this was reversed in the previous UM case. In the same case (*case #2*), we found that ODV/VEN was greater than 1, indicating that the case was not PM as reported before [48]. *Case #2* in the present study is below the previous median ODV/VEN (3.1 and 2.7). This discrepancy could be due to different genotypes [CYP2C19 IM/CYP2D6 IM in the present case versus CYP2C19 EM/CYP2D6 EM and CYP2C19 PM/CYP2D6 EM in previous cases] [44]. Arneth et al.’s [54] results agree with our result, as they reported ODV/VEN equal to 1.1 ± 0.8 for CYP2D6 IM.

Citalopram (CIT) is a potent selective serotonin reuptake inhibitor in the central nervous system that is prescribed for the treatment of depression. It is metabolized in the liver through *N*-desmethylation to desmethylcitalopram (DCIT) [55]. It exhibits large inter-individual variations in plasma and a difference in clinical response and toxicity. The primary cause of this variance is thought to be the individual variations in the cytochrome P450 enzyme activity that catalyze the metabolism of CIT. CYP2C19, CYP2D6, and CYP3A4 were found to be the catalytic factors involved in the synthesis of DCIT in some in vitro experiments [56]. According to Sindrup et al. [57], there was a significant correlation between the *CYP2C19* genotype and the *N*-demethylation of CIT. The CIT case in the present study displayed that her genotyping of *CYP2D6* and *3A4* were gNMs and became pPMs due to co-administration of 9-THC in combination with CIT. In addition, her *CYP2C19* genotyping was IM, but it was phenoconverted into pPM due to the administration of CIT. Yu et al. [55] and Faraj et al. [58] confirmed that DCIT/CIT < 0.24 is considered PM. This is in accordance with case *#3* in the present study, which ensures DCIT/CIT could reflect CYP2C19 activity, and if it is less than 0.24, we could predict the CYP2C19 PM phenotype.

Methadone is a mu-opioid receptor agonist that is frequently used in methadone maintenance therapy (MMT) for people with heroin addictions. The wide range of stabilized methadone dosages needed by patients under MMT indicates significant inter-individual heterogeneity in methadone response [59]. The liver is primarily responsible for methadone metabolism. The main biotransformation of methadone is the N-demethylation to 2-ethylidene-1,5-dimethyl-3,3-diphenylpyrrolidine (EDDP) [60]. The methadone–EDDP ratio (MMR) in plasma ranged from 5.6 to 15.1 [59,61,62]. The highest MMR was 32.7, which was reported in a previous death case of methadone toxicity [63]. The methadone case in the present study showed the lowest limit of the previously mentioned range of the MMR. McCarthy et al. suggested categorizing the metabolic state of subjects in relation to the MMR [PM ≥ 16, IM 12 to <16, NM 5 to <12, and UM < 5] [64]. With the application of the previously mentioned suggestion in our case, the MMR was 5.6, so it is expected to be NM. The *CYP2D6* genotyping revealed that the case is IM and became PM due to PC [2 inhibitors were detected in the blood (paroxetine and diazepam)]. The discrepancy could be due to the complexity of methadone metabolism. According to current knowledge, a number of CYP 450 enzyme systems (such as CYP3A4, 2B6, 2C19, 2D6, 2C9, and 2C8) probably contribute to the N-demethylation of methadone to EDDP [65,66]. As a result, different people may have distinct metabolic pathways [65]. Understanding how genes affect methadone metabolism is still a relatively new field of study. Victorri-Vigneau et al. proposed that polymorphisms in *CYP2B6* might in fact influence the rate of methadone metabolism, while polymorphisms in *CYP2D6* seem not to affect it [66].

## 5. Conclusions

In conclusion, the results of the present study revealed a statistically significant rate of PC for the three investigated enzymes, with a higher frequency of PMs after PC. Compatibility was seen between the results of the genomic evaluation after PC and the observed MRs of venlafaxine, citalopram, and fentanyl. Thus, the present study highlights that the actual phenotype cannot be deduced with certainty due to the possible existence of a significant PC, demonstrating the idea that DDGIs should be considered in both clinical and *post-mortem* contexts. Further research is necessary to enable a more accurate presentation of toxicogenetics testing in routine forensic casework.

## Figures and Tables

**Table 1:** Details of target and probe in the HaloPlex Target Enrichment System.

Target ID	Interval	Regions	Size	Coverage	High Coverage	Low Coverage
*CYP2D6*	chr22:42522490-42540497	10	2810	69.64	7	3
*CYP2C19*	chr10:96447900-96613042	15	4268	100	15	0
*CYP3A4*	chr7:99354572–99381913	14	3698	99.49	14	0

**Table 2:** Distribution of cases according to their sex in relation to mode of death (MoD).

Sex	MoD			Total
	Accidental	Suicidal	Other	
Females	10	5	1	16
Males	25	0	4	29
Total	35	5	5	45

**Table 3:** *CYP2D6*, *2C19*, and *3A4* allele functions identified in the present study.

CYP2D6	Function
*1	Normal function
*3 (rs35742686)	No function
*4 (rs3892097)	No function
*5 (deletion)	No function
*6 (rs5030655)	No function
*9 (rs5030656)	Decreased function
***CYP2C19***	**Function**
*1	Normal function
*2 (rs4244285)	No function
*35 (rs17885098, rs4417205, rs3758581)	No function
***CYP3A4***	**Function**
*1	Normal function
*22 (rs35599367)	Decreased function

*: It is part of allele name.

**Table 4:** Frequencies of *CYP2D6*, *CYP2C19,* and *CYP3A4* genotypes and the corresponding g-phenotype.

*CYP2D6* Genotype	Frequency	g-Phenotype
*1 × 2/*1	2(4.4)	gUM
*1 × 3/*4	1(2.2)	gUM
*1 × 2/*4	1(2.2)	gNM
*1/*1	26(57.8)	gNM
*1/*3	1(2.2)	gIM
*1/*4	8(17.8)	gIM
*1/*9	1(2.2)	gIM
*3/*5	1(2.2)	gPM
*4/*4	2(4.4)	gPM
*4/*5	1(2.2)	gPM
*4/*6	1(2.2)	gPM
***CYP2C19* genotype**	**Frequency**	**g-Phenotype**
*1/*35	31(68.9)	gIM
*2/*35	14(31.1)	gPM
***CYP3A4* genotype**	**Frequency**	**g-Phenotype**
*1/*1	38(84.4)	gNM
*1/*22	7(15.6)	gIM
Total	45(100)	-

g-phenotype: genotypic phenotype. *: It is part of allele name.

**Table 5:** Phenotypes and metabolic ratio (MR) in drug-related deaths compared with previous studies.

Case	*CYP2D6*			*CYP2C19*			*CYP3A4*			*MR*
	Gen	g-p	p-p	Gen	g-p	p-p	Gen	g-p	p-p	
*#1*	*1/*4	IM	PM	*1/*35	IM	PM	*1/*1	NM	NM	VEN/ODV 256/130
*#2*	*1/*1	NM	IM	*1/*35	IM	IM	*1/*1	NM	IM	VEN/ODV 13/20 Fen/Nfen 5/0.6
*#3*	*1/*1	NM	PM	*1/*35	IM	PM	*1/*1	NM	PM	CIT/DCIT 2145/359
*#4*	*1/*4	IM	PM	*1/*35	IM	IM	*1/*1	NM	PM	M/EDDP 252/45

*: It is part of allele name. Gen: genotype; g-p: genotypic phenotype; p-p: phenotype after phenoconversion; NM: normal metabolizer; IM: intermediate metabolizer; PM: poor metabolizer; MR: metabolic ratio; VEN/ODV: venlafaxine/O-desmethylvenlafaxine; Fen/Nfen: fentanyl/nor-fentanyl; CIT/DCIT: citalopram/N-desmethylcitalopram; M/EDDP: methadone/2-ethylidene-1,5-dimethyl-3,3-diphenylpyrrolidene.

## Data Availability

All data generated or analyzed during this study are included in this published article. For any additional requests, the datasets generated during and/or analyzed during the current study are available from the corresponding author upon reasonable request.
